# Antibodies to Laminin β4 in Pemphigoid Diseases: Clinical–Laboratory Experience of a Single Central European Reference Centre

**DOI:** 10.3390/antib14030066

**Published:** 2025-08-01

**Authors:** Maciej Marek Spałek, Magdalena Jałowska, Natalia Welc, Monika Bowszyc-Dmochowska, Takashi Hashimoto, Justyna Gornowicz-Porowska, Marian Dmochowski

**Affiliations:** 1 Autoimmune Blistering Dermatoses Section, Department of Dermatology, Poznan University of Medical Sciences, 60-355 Poznan, Poland; 2 Doctoral School, Poznan University of Medical Sciences, 60-812 Poznan, Poland; 3 Cutaneous Histopathology and Immunopathology Section, Department of Dermatology, Poznan University of Medical Sciences, 60-355 Poznan, Poland; 4 Department of Dermatology, Graduate School of Medicine, Osaka Metropolitan University, Osaka 545-8585, Japan; 5 Department and Division of Practical Cosmetology and Skin Diseases Prophylaxis, Poznan University of Medical Sciences, 60-355 Poznan, Poland

**Keywords:** pemphigoid anti-p200, laminin β4, autoimmune blistering diseases, direct immunofluorescence

## Abstract

**Background/Objectives:** Anti-p200 pemphigoid is a rare and likely underdiagnosed autoimmune blistering disorder. Laminin γ1 and laminin β4 have been implicated as potential target antigens in its pathogenesis. Recently, a novel indirect immunofluorescence assay targeting anti-laminin β4 antibodies has been developed, demonstrating high sensitivity and specificity, and offering a valuable tool for improved diagnosis. **Methods:** Of the 451 patients, 21 were selected for further laboratory analysis based on medical records. Sera from 10 patients, which showed a positive direct immunofluorescence (DIF) result and negative results in multiplex enzyme-linked immunosorbent assays (ELISAs) and/or mosaic six-parameter indirect immunofluorescence (IIF) for various autoimmune bullous diseases, were tested for the presence of anti-laminin β4 antibodies. Additionally, sera from 11 patients with positive DIF and positive ELISA for antibodies against BP180 and/or BP230 were analyzed. **Results:** Among the 10 patients with positive DIF and negative ELISA and/or mosaic six-parameter IIF, 6 sera were positive for anti-laminin β4 antibodies. These patients presented with atypical clinical features. In contrast, all 11 sera from patients with both positive DIF and positive ELISA for BP180 and/or BP230 were negative for anti-laminin β4 antibodies. **Conclusions:** In patients with a positive DIF result but negative ELISA and/or mosaic six-parameter IIF findings, testing for anti-laminin β4 antibodies should be considered. Furthermore, in cases presenting with atypical clinical features—such as acral distribution of lesions, intense pruritus, or erythematous–edematous plaques—the possibility of anti-p200 pemphigoid should be included in the differential diagnosis.

## 1. Introduction

Among various autoimmune bullous diseases (AIBDs), subepidermal autoimmune bullous dermatoses, with bullous pemphigoid (BP) being the most prevalent subtype, represent a group of disorders characterized by considerable clinical heterogeneity. While the classic presentation involves tense bullae and post-bullous erosions, less obvious manifestations, such as eczematous, erythematous–edematous, or urticarial lesions, may also occur. Pruritus of varying intensity may also be observed in BP presenting with an atypical clinical course. Accurate diagnosis is essential for selecting appropriate therapeutic strategies and determining patient prognosis [1,2].

The dermal–epidermal junction (DEJ) is a highly specialized basement membrane zone that maintains skin integrity by anchoring basal keratinocytes to the underlying dermis. It is composed of a complex interplay of structural proteins, including transmembrane and extracellular matrix components [3,4]. BP180 (collagen XVII) is a transmembrane protein that extends through the plasma membrane of basal keratinocytes, forming part of the hemidesmosomal complex and connecting to the extracellular laminin 332. Intracellularly, BP230 binds the cytoplasmic tail of BP180 and links it to the keratin cytoskeleton, ensuring structural resilience [5]. Laminin β4, together with laminin α6, forms laminin 332, which plays a central role in connecting the basal keratinocytes to the dermis by interacting with integrin α6β4 and type VII collagen anchoring fibrils [6]. Laminin γ1, in contrast, is a more broadly distributed laminin subunit incorporated into multiple laminin trimers beyond just laminin 332 [7,8]. Laminin β4 appears to be more directly involved in DEJ stability and hemidesmosome function, while the role of laminin γ1 remains less defined and possibly secondary in pathogenicity [9]. It is possible that the specific autoantigen targeted—BP180, BP230, laminin β4, or γ1—plays a role in shaping clinical manifestations, histopathological patterns, and treatment outcomes in pemphigoid disorders.

The gold standard in the diagnosis of pemphigoid-related diseases remains direct immunofluorescence (DIF) of perilesional skin, which reveals linear deposits of IgG, IgA, or C3 along the DEJ. Techniques that support the diagnostic process include mosaic six-parameter indirect immunofluorescence (IIF) and multiparametric enzyme-linked immunosorbent assay (ELISA), which detect the six most common autoantibodies found in various AIBDs [10]. Other autoantibodies detected in the diagnosis of blistering diseases include those directed against laminin 332, laminin γ1, integrin α6, and integrin β4 [11].

BP180 and BP230 are the major autoantigens in BP. Serological detection of autoantibodies against BP180 and BP230 is therefore central to the diagnosis of pemphigoid diseases [12,13]. The use of multiplex ELISA and mosaic IIF panels typically includes these antigens to help confirm a diagnosis. However, some patients show positive DIF findings but lack detectable antibodies to BP180/BP230, suggesting the involvement of alternative autoantigens such as laminins. Identifying such cases is crucial for understanding atypical presentations and refining diagnostic pathways [14,15].

Anti-p200 pemphigoid is a rare AIBD named for the presence of circulating autoantibodies targeting a 200 kDa protein localized to the lower portion of the lamina lucida of the basement membrane zone [16]. Clinically, the disease exhibits a highly heterogeneous presentation and may resemble other AIBDs, such as BP, inflammatory epidermolysis bullosa acquisita, linear IgA disease, or herpetiform pemphigus [16]. There are also reports of anti-p-200 pemphigoid with similarity to non-AIBD dermatoses, such as erythema multiforme or acquired reactive perforating collagenosis [17,18]. In patients with anti-p200 pemphigoid, lesions most commonly appear on the limbs and trunk in the form of blisters or vesicles, and less frequently as erythematous–edematous lesions [16].

Anti-p200 pemphigoid was proposed to be termed anti-laminin γ1 pemphigoid, when approximately 90% of patient sera were shown to react with recombinant protein of laminin γ1 [19]. However, a subsequent study reported reactivity with a recombinant protein for a fragment of laminin γ1 in only 69% of cases, indicating that this antigen is not universally present among patients with anti-p200 pemphigoid [20]. Furthermore, both ex vivo and in vivo studies failed to confirm a direct pathogenic role for laminin γ1, prompting further investigation into alternative target antigens [9]. More recently, the β4 subunit of laminin has been identified as a potential autoantigen, with anti-laminin β4 antibodies detected in 100% (60/60) of tested patients with anti-p200 pemphigoid [21]. Furthermore, emerging evidence indicates that laminin β4, rather than laminin γ1, may play a central role in the blister formation observed in patients with anti-p200 pemphigoid [9].

The clinical presentation of anti-p200 pemphigoid is heterogeneous, and its pathogenesis remains poorly understood. Most published cases originate from Japan, likely reflecting regional research activity and reporting trends [16]. Given its underdiagnosis, especially outside East Asia, new clinical and laboratory data are essential. To date, only two cases have been reported in Poland, underscoring the need for further investigation in this population [22,23].

The aim of our study was to analyze sera for the presence of anti-laminin β4 antibodies in patients with positive DIF and negative results in multiplex ELISA and mosaic six-parameter IIF, in comparison to patients with positive DIF and positive multiplex ELISA for BP180 and/or BP230.

## 2. Materials and Methods

The study was carried out at a tertiary referral center specializing in AIBDs, within the dermatology department of a central European university.

Patients with IgA deposition on DIF were excluded because the anti-laminin β4 ELISA used in this study is designed to detect IgG and IgG4. The presence of IgA at the dermo-epidermal junction suggests alternative disease mechanisms (e.g., IgA pemphigoid or linear IgA disease), which fall outside the scope of our investigation focused on IgG-mediated pemphigoid variants. Similarly, we excluded patients with mucosal involvement or mucosal biopsy samples to avoid overlap with mucous membrane pemphigoid (MMP) or pemphigus, and to ensure diagnostic consistency. In clinical practice, mucosal biopsies are also more technically challenging and less frequently performed, which may affect diagnostic comparability.

A preliminary analysis was conducted on data from 451 patients evaluated in our center in the years 2019–2025. Of these, 106 patients exhibiting IgA deposition on DIF or circulating IgA antibodies were excluded. From the remaining cohort of 345 patients, individuals with positive results in IIF on the mosaic six-parameter substrate or multiparametric ELISA were further excluded. This refinement yielded a study population of 47 patients. From this group, we further excluded individuals whose DIF samples were taken from mucosal membranes or who had a clinical history of mucosal involvement, resulting in the exclusion of 11 patients and a final study cohort of 36 individuals. From this group, 10 patients were randomly selected for analysis based on the following inclusion criteria: (1) positive direct immunofluorescence (DIF) showing linear IgG and/or C3 deposition at the DEJ, (2) negative results in multiparametric ELISA and mosaic six-parameter IIF, and (3) no mucosal involvement based on clinical history and biopsy site. Additionally, serum samples from 11 patients with positive DIF findings and seropositivity for BP180 and/or BP230, as determined by multiparametric ELISA, were included as a control. Five mL of blood serum was obtained from each participant. The samples were centrifuged for 10 min at 3500 rpm. All serum samples were stored at either −80 °C or −20 °C until analysis.

All patients whose sera were analyzed underwent comprehensive clinical evaluation, including detailed documentation of the morphology and distribution of skin lesions. Additionally, a structured interview was conducted to assess subjective symptoms associated with the cutaneous manifestations. Importantly, none of the patients had received treatments for AIBDs prior to undergoing skin biopsy for DIF analysis.

The study was conducted in accordance with the Declaration of Helsinki and approved by the Ethics Committee of the Poznan University of Medical Sciences (protocol no. 540/13). Written informed consent was obtained from all participants for inclusion in the study and for publication of the anonymized data presented herein.

### 2.1. Direct Immunofluorescence Procedure

In this case, DIF analysis of perilesional skin tissue was conducted to detect not only the commonly assessed immunoreactants—IgA, IgM, IgG, and complement component C3—but also the IgG subclasses IgG1 and IgG4 [24]. Based on the experience of our laboratory and other corroborated research groups, IgG4 deposition patterns often provide clearer, more interpretable images compared to those obtained with standard immunoglobulins such as total IgG [25].

For staining, 4 μm thick cryostat sections were prepared from perilesional skin samples. These sections were incubated for 30 min at room temperature in a humidified chamber with fluorescein isothiocyanate (FITC)-labeled antibodies. For DIF, separate FITC-conjugated polyclonal rabbit antibodies against human IgG (cat. no. F0202), IgA (cat. no. A0262), IgM (cat. no. F0254), and C3c (cat. no. A0062) were used (Agilent Technologies, formerly Dako, Glostrup, Denmark), according to the manufacturer’s protocol, as well as FITC-conjugated mouse monoclonal antibodies targeting human IgG1 and IgG4 (clone HP-6025, Sigma-Aldrich, St. Louis, MO, USA). All antibodies were diluted 1:100 in phosphate-buffered saline (PBS).

Following incubation, slides were rinsed in PBS (pH 7.2) at room temperature for 15 min with gentle shaking. After washing, the sections were mounted with coverslips and examined independently by two observers to reduce interpretative bias. Imaging was performed using a fluorescence microscope equipped with a short-arc mercury lamp (BX40, Olympus, Tokyo, Japan). Staining intensity was evaluated using a semi-quantitative four-point scale (ranging from “−” to “+++”) at an original magnification of 40×.

### 2.2. Multiplex ELISA Procedure

We employed the Euroimmun Dermatology Profile ELISA kit (Euroimmun, Lübeck, Germany), a commercially available multiplex assay designed to detect serum IgG autoantibodies. The assay included six distinct antigens, BP180, BP230, desmoglein 1 (DSG1), desmoglein 3 (DSG3), envoplakin, and type VII collagen, each coated in individual wells [10]. The results were interpreted semi-quantitatively, using the manufacturer’s recommended cut-off ratio of 1 for positivity.

Serum samples were diluted 1:101 in PBS containing 0.1% (*w*/*v*) casein and incubated on the antigen-coated wells for 30 min at room temperature. After washing, bound IgG antibodies were detected using a peroxidase-conjugated anti-human IgG secondary antibody (Euroimmun, Lübeck, Germany), followed by a 15 min color development step with tetramethylbenzidine substrate. Absorbance was measured at 450 nm with a 620 nm reference filter using an automated microplate reader (Lededect 96, Dynamica GmbH, Salzburg-Mayrwies, Austria).

All assays were performed using ELISA plate readers (Asys Expert 96, Biochrom Ltd, Cambridge, UK) or Ledetect 96, Dynamica GmbH, Salzburg-Mayrwies, Austria) operated with MikroWin 2000 version 4.4 software. A single trained technician conducted all measurements in accordance with the manufacturer’s protocol.

### 2.3. Mosaic Laminin β4 Indirect Immunofluorescence Procedure

To detect anti-laminin β4 antibodies, we employed an IIF assay based on Biochip™ technology (Euroimmun, Lübeck, Germany), incorporating recombinant HEK293 cells transfected with laminin subunit β4 gene and control non-transfected EU90 cells. This assay employed secondary antibodies against total human IgG, supplemented with anti-IgG4, to enhance detection sensitivity and isotype coverage (Euroimmun, Lübeck, Germany). The assay was performed using recommended serum dilutions of 1:10 and 1:100 [21,26].

Specific staining is characterized by a smooth to fine granular cytoplasmic fluorescence in subunit β4-expressing cells, occasionally with membrane accentuation and, depending on the sample, nuclear involvement. Control cells (EU90) remain non-reactive in positive cases, allowing for clear discrimination. If both transfected and control cells exhibit fluorescence, non-specific antibody reactivity is suspected. To minimize the risk of technical errors, all analyses were performed in duplicate by a single operator.

This assay has demonstrated a sensitivity of 99.2% and a specificity of 99.3%, as reported in a validation study involving 239 sera from patients with confirmed anti-p200 pemphigoid and 300 healthy control sera. Sensitivity was established by identifying 237 sera that showed specific binding to recombinant laminin β4-transfected HEK293 cells, while specificity was based on only 2 out of 300 healthy donor samples showing weak reactivity. All samples were tested using a standardized IIF protocol incorporating internal controls (non-transfected EU90 cells) to distinguish specific from non-specific staining. Diagnosis of anti-p200 pemphigoid in the patient group had been previously confirmed by immunoblotting with dermal extracts, ensuring reference-standard classification for sensitivity analysis [26].

## 3. Results

The group of patients with positive DIF and negative results in additional diagnostic tests included five women and five men, with a mean age of 79.0 ± 11.7 years. Six patients tested positive for anti-laminin β4 antibodies using the IIF assay, with two showing positivity at a dilution of 1:100 and four at a dilution of 1:10. The remaining four patients were negative for anti-laminin β4 antibodies. In all patients positive for anti-laminin β4 antibodies by IIF, DIF revealed IgG4 deposition with a staining intensity of at least ++, and the clinical presentation of skin lesions was polymorphic. All patients reported pruritus for the skin lesions, with varying intensity.

Detailed information regarding DIF results, anti-laminin β4 IIF findings, and clinical presentation is provided in Table 1.

**Figure 1:**
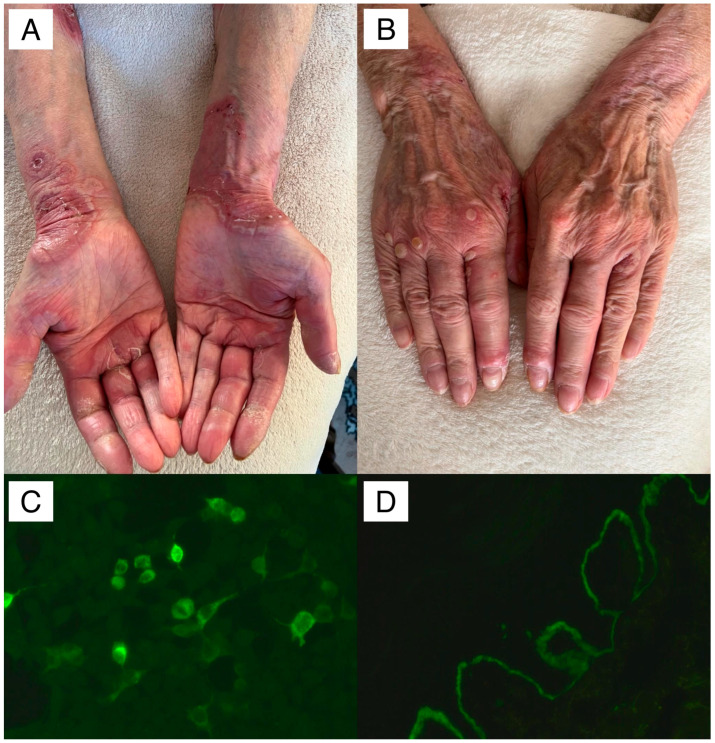
An 82-year-old patient presented with linear deposits of IgG1 (+), IgG4 (++), and C3 (++) along the DEJ on DIF, and tested negative in both multiparametric ELISA and mosaic six-parameter IIF. (**A**) Disseminated vesicles, erythematous-desquamative lesions, and crusts arranged in an annular pattern resembling erythema multiforme on the palmar surfaces of the hands and forearms. (**B**) Disseminated blisters, vesicles, and crusts on the dorsal surfaces of the hands. (**C**) BIOCHIP IIF revealing positive reaction of IgG and IgG4 autoantibodies with recombinant HEK293 cells transfected with laminin subunit β4 gene (original objective magnification ×40). (**D**) Linear deposits of IgG4 (++) along the DEJ on DIF visualized using a short-arc mercury lamp-operated microscope (Olympus, Tokyo, Japan) (original objective magnification ×40).

Clinical photographs of patient #1, along with the corresponding anti-laminin β4 IIF result and DIF showing linear IgG4 (++) deposits along DEJ, are presented in Figure 1.

The control group of patients with positive DIF and positive ELISA for BP180 and/or BP230 included six women and five men, with a mean age of 78.8 ± 5.38 years. All patients tested negative for anti-laminin β4 antibodies using the IIF assay.

Detailed information for the control group, regarding DIF results, ELISA results, and anti-laminin β4 IIF findings, is presented in Table 2.

Representative IIF images are shown in Supplementary Figure S1.

## 4. Discussion

We designed a study in which we analyzed a group of patients with a positive DIF result, without positive findings in ELISA, and without mucosal involvement. During patient selection, 36 such cases were identified out of 451, representing nearly 8% of the cohort. In a study conducted by Giurdanella et al., 5% of patients with pemphigus showed a positive DIF result in the absence of a positive ELISA result [27]. Although such patients represent a minority among individuals with AIBDs, this still corresponds to dozens of cases without a definitive diagnosis. In this study, we also excluded patients with mucosal involvement, as DIF is primarily performed in the context of pemphigus diseases or MMP, and mucosal biopsy is technically more challenging in clinical practice compared to skin biopsy [28,29].

BP may present atypically with eczematous or urticarial lesions, or with blisters located on acral areas, which can make it difficult to distinguish from anti-p200 pemphigoid based solely on clinical appearance [30,31]. There are reports in the literature describing the development of atypical forms of BP following treatment with linagliptin and vildagliptin, in which the diagnosis was based on DIF and histopathological examination of the skin, without confirmation by ELISA [32,33]. In light of recent findings, such cases may warrant an extended diagnostic work-up and differentiation from anti-p200 pemphigoid. In our group of patients with positive ELISA results for BP180 and/or BP230, none of the serum samples showed the presence of antibodies against laminin β4. This demonstrates that multiparametric ELISA is a highly valuable diagnostic tool, particularly useful for confirming atypical presentations of BP and differentiating them from anti-p200 pemphigoid. We recommend its use as the initial diagnostic step, followed—if necessary—by IIF for the detection of antibodies against laminin β4 [10,21].

The diagnosis of anti-p200 pemphigoid is primarily supported by the detection of serum antibody reactivity against a 200 kDa protein (p200) in human dermal extracts using immunoblotting [34]. Rasheed proposed that the diagnostic work-up for anti-p200 pemphigoid should include serration pattern analysis in DIF and IIF on salt-split skin, with the final diagnosis based on immunoblotting for the 200 kDa protein [35]. However, in clinical practice, this approach is challenging due to the limited availability of immunoblotting, which is restricted to specialized centers. With the discovery that patients with anti-p200 pemphigoid may produce antibodies against laminin γ1 and β4, van Beek et al., in a 2024 review, proposed more liberal diagnostic criteria for anti-p200 pemphigoid. Based on the current body of evidence and prior reports, the revised laboratory criteria include the presence of antibodies against laminin β4 or γ1, or detection of the p200 protein by immunoblotting, or IgG reactivity with the blister floor on salt-split skin by indirect immunofluorescence microscopy [16,36]. The broad acceptance of various laboratory tests in the diagnosis of anti-p200 pemphigoid stems from the fact that its exact pathogenesis remains unknown, and the roles of laminin γ1 and β4 are still under investigation [37]. In 2025, Koga et al. also suggested that anti-laminin β4 antibodies, rather than anti-laminin γ1, play a more significant role in blister formation [38]. On the other hand, authors noticed that the absence of laminin β4 expression in murine skin may indicate that its role in human skin is less substantial than previously thought [21,38]. Considering all of these findings and the lack of definitive diagnostic criteria for anti-p200 pemphigoid, we chose to use the term anti-laminin β4 pemphigoid in the title of our study.

In our study, six out of ten tested serum samples were positive for anti-laminin β4 antibodies. Among these patients, 66.7% were male, and the mean age was 79.0 ± 11.78 years. Blisters or vesicles were observed in four out of six patients, while urticarial lesions were present in two out of six. All patients had lesions on the extremities, and in three cases, the trunk was also involved. In a review by Kridin analyzing 113 cases of anti-p200 pemphigoid, 75.2% of patients were male, and the mean age at onset was 65.5 years—lower than that typically observed in BP. All patients presented with blisters or vesicles and urticarial plaques were the second most common manifestation, occurring in just over half of the cases. Skin lesions most commonly involved the extremities and trunk, with palms and soles affected in just over half of the patients [16]. Similarly to our patient group, the majority of cases were male; however, the mean age of disease onset was higher, and not all patients developed blisters or vesicles. In Kridin’s analysis, most patients were of Japanese origin, among whom mucosal involvement was significantly less frequent than in other populations (22.5% vs. 48.2%; *p* < 0.001). This raises the possibility that other clinical differences may exist between populations, although further studies in diverse cohorts are needed to confirm such trends. Our own findings should also be interpreted in the context of a relatively small sample size.

It is worth highlighting the case of elderly patient #1, who presented with skin lesions resembling erythema multiforme. To date, two cases of anti-p200 pemphigoid with erythema multiforme-like features have been described in the literature: a 26-year-old man (negative for anti-laminin γ1 antibodies) and a 48-year-old woman (not tested for anti-laminin γ1 antibodies). Both had mucosal involvement and widespread skin lesions, which differs from the more localized and mucosa-sparing presentation observed in our patient [17,39]. Further research into the roles of laminin β4 and laminin γ1 may help clarify the clinical variability of anti-p200 pemphigoid.

A case of anti-p200 pemphigoid with exclusive IgG deposits on DIF has been described in the literature [40]. Patient #2 in our study exhibited atypical skin lesions resembling folliculitis and showed exclusively IgG4 deposits (++) on DIF, confirmed twice from different skin samples collected at different time points. One of the theories proposed by Dainichi et al. suggests that IgG4 antibodies may be associated with the atypical, noninflammatory phenotype of BP, characterized by the absence of urticarial lesions preceding blister formation [41]. Our patient #2 was the only case without blisters or urticarial eruptions and presented with the most atypical clinical features. All patients with positive IIF results for laminin β4 antibodies demonstrated IgG4 deposits (++) on DIF. In contrast, the remaining five patients also exhibited C3 deposits in addition to IgG4. This observation may support the hypothesis that IgG4 is associated with a noninflammatory phenotype of BP; however, further studies are needed to confirm this relationship. Additionally, the IIF laminin β4 antibody assay detects both total IgG and IgG4-specific antibodies, which may aid in the diagnosis of patients with IgG4 deposits on DIF. Nevertheless, this requires further investigation.

The main limitation of our study is the small sample size, which reflects the rarity of AIBDs, and more specifically, the limited number of patients with positive DIF but negative ELISA results. Another limitation is that only antibodies against laminin β4 were assessed, without parallel testing for laminin γ1 or reactivity with the 200 kDa p200 protein using immunoblotting with dermal extracts. However, the assay for anti-laminin β4 antibodies is widely available, in contrast to the other two methods, which remain largely restricted to specialized laboratories.

## 5. Conclusions

Given cost-effectiveness considerations, we propose a stepwise diagnostic strategy for AIBDs associated with autoimmunity to non-enzymatic antigens. The initial assessment should include a thorough clinical evaluation of lesion morphology and distribution (“what and where” approach), DIF for identifying IgG1 and IgG4 deposition, and a multiplex six-parameter ELISA for detecting serum IgG autoantibodies. In cases where DIF is positive—indicating non-IgA-mediated autoimmunity—but multiplex ELISA yields negative results across all parameters, we recommend performing IIF for IgG+IgG4 antibodies targeting laminin β4 to enhance diagnostic accuracy.

## Figures and Tables

**Table 1:** Demographic, clinical, and laboratory data of patients with positive DIF results and negative findings in ELISA.

Patient	Gender/Age (Years)	DIF Findings	IIF for Laminin β4	Clinical Presentation
1	F/82	Linear deposits of IgG1 (+), IgG4 (++), and C3 (++) along the DEJ	1:100	Predominantly acral vesicles and blisters in an erythema multiforme-like arrangement
2	M/61	Linear deposits of IgG4 (++) along the DEJ	1:10	Disseminated papulopustular, folliculitis-like lesions on the trunk, back, and extremities
3	F/78	Linear deposits of IgG1 (+), IgG4 (+++), and C3 (+++) along the DEJ	1:10	Tense blisters and erosions distributed across the entire body, including acral regions
4	M/93	Linear deposits of IgG1 (+), IgG4 (++), and C3 (+++) along the DEJ	1:10	Urticarial plaques predominantly on the trunk and lateral aspects of the thighs
5	M/71	Linear deposits of IgG4 (++) and C3 (+++) along the DEJ	1:10	Disseminated erosions covered with brownish crusts, mainly on flexural aspects of the limbs and soles of the feet
6	M/89	Linear deposits of IgM (+), IgG1 (+), IgG4 (++), and C3 (+++) along the DEJ	1:100	Acral blisters and urticarial lesions on the scrotum and medial aspects of the thighs
7	F/68	Linear deposits of IgG1 (+) and C3 (+++) along the DEJ	Negative	Erosions with crusts predominantly on the scalp; few erosions on the trunk
8	F/90	Linear deposits of IgG1 (++) and C3 (+++) along the DEJ	Negative	Isolated erosions on the scalp, with multiple erosions primarily on the trunk and to a lesser extent on the upper extremities
9	F/91	Linear deposits of IgG1 (+/−), IgG4 (+), and C3 (++) along the DEJ	Negative	Disseminated, well-tensed blisters on the trunk and extremities, with scattered erosions
10	M/67	Linear deposits of C3 (++) along the DEJ	Negative	Erythematous and erosive lesions with well-tensed blisters on the extremities, and to a lesser extent on the trunk

DIF, direct immunofluorescence; IIF, indirect immunofluorescence; DEJ, dermal–epidermal junction.

**Table 2:** Demographic and laboratory data of patients with positive DIF results and positive ELISA for BP180 and/or BP230.

Patient	Gender/Age (Years)	DIF Findings	ELISA Results (BP180 and BP230)	IIF for Laminin β4
1	F/70	Linear deposits of C3 (+++) along the DEJ	BP180: 7.54; BP230: 3.42	Negative
2	F/81	Linear deposits of IgG4 (+) and C3 (++) along the DEJ	BP180: 9.82; BP230: 5.82	Negative
3	M/80	Linear deposits of IgG1 (+), IgG4 (++), and C3 (++) along the DEJ	BP180: 1.02	Negative
4	F/78	Linear deposits of IgG4 (+) and C3 (+++) along the DEJ	BP180: 10.72	Negative
5	M/86	Linear deposits of IgG4 (+) and C3 (++) along the DEJ	BP180: 11.44	Negative
6	M/71	Linear deposits of IgG4 (+) and C3 (++) along the DEJ	BP180: 8.33	Negative
7	M/80	Linear deposits of IgG (+), IgG1 (+++), IgG4 (+), and C3 (+) along the DEJ	BP180: 7.03	Negative
8	F/80	Linear deposits of IgG4 (++) and C3 (++) along the DEJ	BP180: 7.19	Negative
9	M/73	Linear deposits of C3 (+++) along the DEJ	BP180: 8.03	Negative
10	F/85	Linear deposits of IgG (+), IgG4 (+), and C3 (++) along the DEJ	BP180: 5.35; BP230: 1.29	Negative
11	F/83	Linear deposits of IgG (+), IgG1 (+), IgG4 (+), and C3 (++) along the DEJ	BP180: 8.95; BP230: 5.00	Negative

DIF, direct immunofluorescence; IIF, indirect immunofluorescence; DEJ, dermal–epidermal junction; ELISA, enzyme-linked immunosorbent assay. ELISA results are expressed as semi-quantitative ratio values (OD_sample/OD_calibrator × lot-specific factor), calculated according to manufacturer’s instructions (Euroimmun). A ratio of ≥1.0 is considered positive.

## Data Availability

All data are available upon request.

## Supplementary Materials

The following supporting information can be downloaded at: https://www.mdpi.com/article/10.3390/antib14030066/s1. Figure S1: Mosaic indirect immunofluorescence for anti-laminin β4 IgG+IgG4 antibodies.
