# Supplementary material for: Antibodies to Laminin β4 in Pemphigoid Diseases: Clinical–Laboratory Experience of a Single Central European Reference Centre
Source: Antibodies (Basel). 2025 Aug 1;14(3):66. doi: 10.3390/antib14030066 (PMC12371968; doi:10.3390/antib14030066)
Supplement: Supplementary file 1 [file antibodies-14-00066-s001.zip › antibodies-3757957-supplementary.pdf]

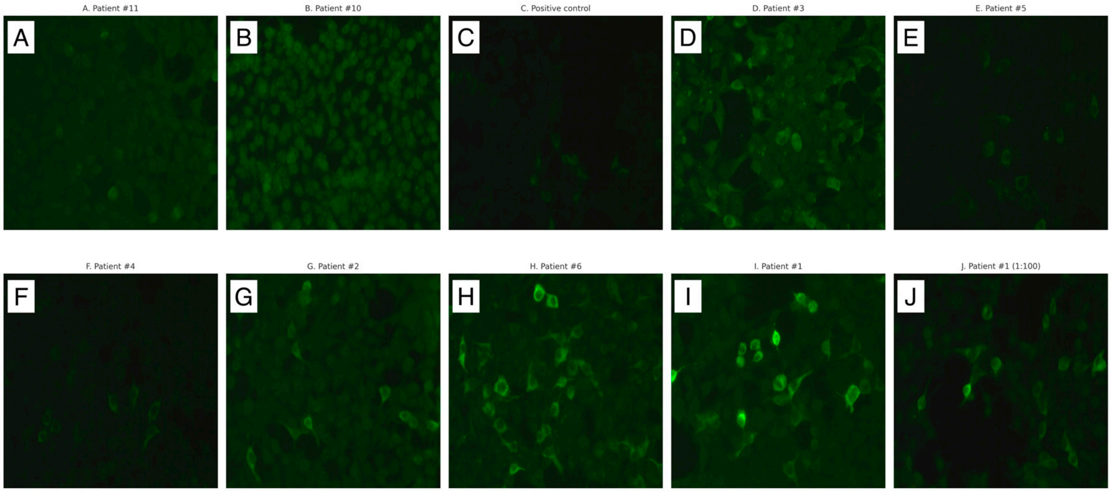

Supplementary Figure S1. Mosaic indirect immunofluorescence for anti-laminin β4 IgG+IgG4 antibodies. (A–B) Negative IIF (1:10 dilution) in Patient #11 and #10 with positive BP180 ELISA but no laminin β4 reactivity. (C) Positive control serum provided by the manufacturer. (D–I) Positive IIF (1:10) in Patients #3, #5, #4, #2, #6, and #1. (J) Positive IIF at 1:100 dilution in Patient #1. All images were acquired at 40× magnification. Results and endpoint dilutions are detailed in Tables 1 and 2.
